# The Decision Process Scale (DPS): Self-report measures of reliance on rules, cost–benefit reasoning, intuition, and deliberation in (moral) decision-making

**DOI:** 10.3758/s13428-025-02933-7

**Published:** 2026-02-13

**Authors:** Vanessa Cheung, Maximilian Maier, Falk Lieder

**Affiliations:** 1 Department of Experimental Psychology, University College London, London, England; 2 Behavioural Science Group, Warwick Business School, University of Warwick, Coventry, England; 3 Max Planck Institute for Intelligent Systems, Tübingen, Germany; 4 Department of Psychology, University of California, Los Angeles, CA USA

**Keywords:** Moral dilemmas, Moral decision-making, Utilitarianism, Deontology, Process measures, Decision-making styles, Intuition versus deliberation

## Abstract

Understanding how people make decisions in specific situations is a central challenge in (moral) psychology research. Yet there are no existing self-report scales for measuring the process of decision-making in individual dilemmas (as opposed to general moral attitudes or beliefs about moral decision-making). We address this gap by devising new self-report measures of several of the processes by which people make moral decisions and validate them using realistic moral dilemmas, including six new vignettes that we developed. The resulting 12-item Decision Process Scale (DPS) can be used to measure how much people rely on rules versus cost–benefit reasoning and how much they rely on intuition versus deliberation in the specific moral dilemmas they face in a laboratory experiment or in the real world.

Research on morality is one of the fastest-growing fields of psychology (Priva & Austerweil, 2015). Some of the field’s open questions include: How do people make moral decisions, and how does this depend on the situation and the person’s prior experience? When do people follow moral rules, and when do they rely on cost–benefit reasoning (CBR)? When do they follow their intuition and gut feelings, and when do they deliberate? Are these two processes opposite ends of a single dimension, or are they better represented by two independent dimensions? Although moral psychology research has made considerable progress on these questions (e.g., Greene and Haidt, 2002; Haidt, 2007; Betsch, 2008; Greene, 2015; Cushman, 2013; Crockett, 2013; Pachur and Spaar, 2015; Bear and Rand, 2016; Gawronski and Ng, 2024; Garrigan et al., 2018; Capraro, 2024; Guzmán et al., 2022; Awad et al., 2018), our understanding of these crucial aspects of moral decision-making is still far from complete. One contribution to closing this gap is to develop standardized measures of the processes by which people make decisions in moral dilemmas, which remains largely unexplored.

Most scales in moral psychology focus on presumably stable individual differences in people’s general beliefs about and attitudes towards morality. According to a recent systematic review by Ni et al. (2023), only two of those measures have sound psychometric properties: the Defining Issues Test (Rest, 1974) and the Oxford Utilitarianism Scale (Kahane et al., 2018). The Defining Issues Test is based on Kohlberg’s theory of moral development (Kohlberg, 1981), and measures what stage of moral development a person is in based on how they justify the actions they consider to be morally right in a predefined set of moral dilemmas. The Oxford Utilitarianism Scale measures people’s endorsement of one major moral theory: utilitarianism.

In addition, the recently developed Deontological-Consequentialist Scale (Mata et al., 2022) can be used to measure people’s endorsement of consequentialism as well as deontology, which are commonly regarded to be two oppositional philosophies. Another measure, the Moral Metacognition Scale (McMahon & Good, 2016), focuses on how much and how well people think about their moral thinking. Further, there has been some work on measures of moral traits in personality psychology, which focus on honesty and humility (Ashton et al., 2014). Related prior work investigated how existing measures of stable personality traits correlate with moral decisions (e.g., Big Five Personality Traits; Luke and Gawronski, 2022).

While the abovementioned studies and scales provide important insights into individual differences in moral decision-making, they do not measure the moral decision-making process. Although there are several self-report measures of people’s decision-making styles (Betsch, 2008; Pachur & Spaar, 2015; Epstein et al., 1996; Bruine de Bruin et al., 2007; Appelt et al., 2011; Scott & Bruce, 1995), they do not capture the unique aspects of moral decision-making as they measure general tendencies rather than the process of a specific decision.

On the other hand, while many experimental studies have investigated the process of how people make moral decisions and found valuable insights (e.g., Oktar et al., 2023), most did not use standardized, validated self-report scales to measure moral decision-making processes. Those who did use validated scales either employed self-report measures of decision-making in general (e.g., Epstein et al., 1996; Bartels, 2008) rather than moral decision-making in a specific moral dilemma, or only measured people’s reliance on different types of moral values (Clifford et al., 2015). These two approaches do not measure the unique moral processes of CBR and following moral rules.

To avoid the limitations of self-report measures, moral psychologists commonly infer the decision process from people’s choices in dilemmas where the theorized mechanisms imply different choices (e.g., Conway and Gawronski, 2013; Gawronski et al., 2017a; Körner et al., 2020). For example, in typical moral dilemmas, the decision mechanism of following the moral rule means not committing sacrificial harm, while sacrificial harm is usually endorsed by the mechanism of cost–benefit reasoning. These methods are confined to moral dilemmas that are designed such that people’s behavior is diagnostic of which decision-making mechanism they rely on. However, in many real-world situations, the different decision mechanisms a person might rely on all recommend the same behavior (Parfit, 2011). Thus, this approach cannot be applied when trying to understand how people make moral decisions in any situation they might encounter in the real world. In addition, the dilemmas are usually designed so that different ethical theories, such as utilitarianism and deontology, lead to opposite choices; however, as we argue in the next section, it is unclear whether this is actually achieved in many dilemmas, and even if it were achieved, it is unclear whether people really think about the decisions in those terms (Kahane, 2015; Bennis et al., 2010).

Other methods for measuring the process of moral decision-making include paradigms that apply eye-tracking and process-tracing methods to moral decision-making (e.g., Ghaffari and Fiedler, 2018; Fiedler and Glöckner, 2015). While these methods reveal which information people consider, they do not directly measure what people do with that information. Moreover, previous studies found that process tracing paradigms can change how people make decisions (Lohse & Johnson, 1996), and eye-tracking is more challenging than administering self-report measures.

Finally, process dissociation approaches have been used to decompose different factors that influence people’s moral decisions, in particular, reliance on consequences, norms, and general preference for inaction (Gawronski et al., 2017a). However, these approaches were not originally developed for use as individual difference measures, and extensions to measure individual differences require a large number of dilemmas to be administered and only show weak and inconsistent relationships with established scales (Körner et al., 2020).

This paper aims to close this crucial gap by providing self-report measures for the processes of moral decision-making. Across three studies, we developed the Decision Process Scale, which measures how much participants reported relying on different processes involved in (moral) decision-making (rules versus CBR, and intuition versus deliberation). In addition, we find that—contrary to prior research that assumed people have to choose between relying on CBR and moral rules (for a review, see Christensen and Gomila, 2012)—the degree to which people rely on CBR and the degree to which they rely on moral rules are two independent dimensions of their decision process. Moreover, we find that the same is also true for the interplay of intuition versus deliberation; while previous work has often construed it as a conflict that has to be resolved in favor of one or the other, some have argued that this is not necessarily the case (Loewenstein et al., 2015). Although these measures were validated using moral dilemmas, they do not rely on any moral concepts within the scale items, making them potentially of interest also to decision-making researchers outside of moral psychology.

## Theoretical background on decision processes

Moral decision-making is often studied in terms of how much people follow moral rules versus rely on CBR, and how much people rely on their moral intuitions versus deliberation. Below, we discuss the literature on these topics and how people combine these different decision mechanisms when making moral decisions.

### Utilitarianism versus deontology

According to deontological moral theories, some actions, such as killing, are always morally wrong, regardless of their consequences (Alexander & Moore, 2021). According to these theories, people should always follow certain moral rules, such as “do no harm.’’ By contrast, consequentialism is the view that whether something is right or wrong depends entirely on its consequences (Sinnott-Armstrong, 2022). Utilitarianism is a special case of consequentialism, which aims to maximize happiness or well-being (Driver, 2022). Previous research in moral psychology usually mapped decision-making in moral dilemmas directly onto the meta-ethical theories of deontology and utilitarianism. For example, the CNI (Consequences, Norms, and Generalized Inaction) model of moral decision-making (Gawronski et al., 2017b) operationalizes utilitarianism as sensitivity to outcomes, and deontology as sensitivity to moral norms.

**Table 1:** Items for the Decision Process Scale (DPS): Reliance on Intuition versus Deliberation and Reliance on Rules versus Consequences Subscales

Intuition	
To what extent did you rely on your intuition when making your decision?	
To what extent did you rely on your emotions when making your decision?	
To what extent did you trust your gut feeling when making your decision?	
Deliberation	
To what extent was it important that you had a rational reason for your decision?	
To what extent did you consider the anticipated consequences of your decision?	
To what extent did you carefully evaluate the pros and cons of your decision compared to the alternative decision?	
Rules	
To what extent was it important that the action you chose did NOT violate any rules?	
To what extent was it important that the action you chose did NOT violate any norms?	
To what extent did you rely on rules when making your decision?	
Consequences	
To what extent was it important that the action you chose would maximize overall happiness for everyone?	
To what extent did you choose your action based on its anticipated consequences?	
To what extent was it important that the action you chose would have better consequences than the action you did not choose?	

*Note.* We presented participants with the prompt “Please consider the following statements and think about to which extent you agree with each statement,’’ to which they indicated their agreement on a scale of 0 (“Not at all’’) to 100 (“Entirely’’)

### Moral rules versus cost–benefit reasoning (CBR)

Moral decision-making is commonly studied using sacrificial dilemmas, where people must decide whether to commit sacrificial harm for the “greater good’’. For instance, a version of the trolley problem tasks the reader with choosing between letting the trolley run over five people or pushing a large man off a footbridge and using his body to stop the trolley (Foot, 1967; Thomson, 1976). Typically, one option is identified as utilitarian, and one as deontological (for a review, see Gawronski and Beer, 2017). Here, the “utilitarian’’ CBR option is to push the large man in front of the trolley (i.e., kill one person to save a greater number of people), whereas the “deontological’’ option is to abstain from it (to follow the moral rule “Do no harm’’).

Although the trolley problem is set up such that the evaluative standard of utilitarianism endorses the action that people would choose if they relied on CBR, it is unclear whether this association also holds in the real world. Human CBR is inevitably fallible because it is computationally intractable to accurately predict and consider *all* possible consequences (Gigerenzer, 2008; Bennis et al., 2010; Gigerenzer, 2010; Maier et al., 2025). Therefore, there is no guarantee that the action a person chooses by CBR will be the one that has the best possible consequences according to utilitarianism.

For example, in a real-world instantiation of the classic trolley problem, the large man’s body could be insufficient to stop the trolley from killing the others (indeed, there is no compelling evidence that a single individual can stop a running trolley). Moreover, there is no guarantee that his body will land on the tracks when you push him. Therefore, it is quite possible that the action recommended by a person’s naive CBR could achieve the negative outcome of killing the man without saving anyone. Even if the man’s body does stop the trolley as intended, there are still many negative indirect effects that are difficult to take into consideration: for instance, that pushing the man would weaken the general moral norm against killing or cause people to fear using footbridges. Thus, following the rule may actually be better from a utilitarian perspective. Further, for participants not trained in moral philosophy, it is unlikely that they have in-depth knowledge about utilitarianism and deontology and explicitly use them as decision mechanisms (Kahane, 2015).

For these reasons, we refer to the two choices by their decision mechanisms, which is “following moral rules’’ versus “relying on cost–benefit reasoning’’ (CBR), and do not equate these to normative theories. Note that CBR here refers to a “first-order’’ or “naive’’ CBR, which compares the numbers of people directly affected by the decision rather than a full cost–benefit analysis, which would also consider all indirect consequences, which is computationally intractable for real-life decisions.

Past research has shown that people are sensitive to a variety of features, such as whether the behavior is an action or an omission (Baron & Ritov, 2004; Yeung et al., 2022; Cheung et al., 2025), the degree of personal force involved (Feltz & May, 2017; Greene et al., 2009), and the extent to which the norms regarding each feature are violated (Nichols & Mallon, 2006). If people were strictly following only one decision mechanism, they should either always choose the CBR option independent of the number of people saved (as long as it is larger than the number of people saved by the alternate option), or always choose the rule option independent of how strongly the norm is violated (Everett & Kahane, 2020). The fact that the employed decision mechanisms vary across situations and are not fixed in that way underscores the importance of measuring decision processes in specific dilemmas.

The Reliance on Rules versus Consequences subscales developed in this article are designed to help researchers investigate how much people rely on CBR versus moral rules in different types of moral decisions (see Table 1). We believe that much of the previous literature discussing utilitarianism versus deontology is best understood as actually discussing reliance on CBR versus rules; therefore, despite their conceptual differences, we validate the proposed measures by comparison to previous scales purporting to measure utilitarianism and deontology.

### Intuition versus deliberation

Previous research on moral decision-making distinguished between intuition and deliberation as two distinct types of cognitive processes (Greene et al., 2008, 2001; Greene, 2013). Intuition, driven by fast, automatic, and emotional responses, is usually linked to following moral rules (e.g., not pushing the man in the trolley problem). By contrast, deliberation involves slow, effortful, reason-based thinking, enabling us to weigh different factors and potential consequences more thoroughly. It is, therefore, linked to CBR. Some previous research has found that reasoning ability and tendency toward deliberative thinking are positively associated with a preference for “utilitarian’’ solutions (Patil et al., 2021; Greene et al., 2008); however, this result is not consistently replicated across different studies and designs (e.g., Rosas and Aguilar-Pardo (2020); Rehren (2024)). Similarly, the relationship between deontology or moral rules and intuition is often small and only found inconsistently (Fahrenwaldt et al., 2025).

Dual-process theories were later extended to reinforcement learning theories of moral decision-making, which link intuition and deliberation to specific types of reinforcement learning (RL) mechanisms that might underlie people’s choices (Cushman, 2013; Crockett, 2013). This RL theory of moral decision-making proposes that intuitive moral rules, such as “do not kill’’, originate from simple, learned action-value associations. The distinctive feature of these value representations is that they are insensitive to the consequences the actions might have in the specific situation in which they are being considered. The RL theory of moral decision-making contrasts these so-called “model-free’’ representations to “model-based’’ moral decision-making, which uses a learned model of how taking different actions in the current situation would affect the state of affairs to reason out which of those choices would produce the best consequences. Cushman (2013) and Crockett (2013) proposed this model-based decision mechanism as a model of CBR and deliberation more generally.

The Reliance on Intuition versus Deliberation subscales developed in this article are designed to help researchers investigate how much people rely on model-free (moral) intuitions versus (model-based) deliberation in different types of moral decisions (see Table 1).

## Scenarios for studying moral decision-making

Moral decision-making has been studied in a variety of contexts, such as realistic field studies (Oktar et al., 2023; Schwitzgebel et al., 2023) and lab experiments, where participants are confronted with moral dilemmas. The most popular task used to study moral decision-making is the trolley problem. However, classic trolley-type dilemmas are often criticized for being unrealistic and bizarre (Bauman et al., 2014; Bennis et al., 2010) due to their complete lack of uncertainty and sometimes absurd situations. Another problem with these dilemmas is that they usually confound the CBR option with action, and the rule option with inaction (Crone & Laham, 2017). For instance, in the footbridge version of the trolley problem, “push the man’’ is both the CBR option and a physical action, whereas the rule option “don’t push the man’’ coincides with inaction (commonly referred to as “omission’’). Because of this confound, a person’s preference for the rule option could simply be a preference for inaction (i.e., omission bias; Baron and Ritov (2004)) rather than a commitment to following moral rules.

To mitigate these limitations, Körner and Deutsch (2023) developed realistic trolley-type dilemmas (which were later revised and adapted by Maier et al. (2025)). Some of them are based on historical events. Importantly, they also dissociate rule-following from inaction and CBR from taking action. We extend this work by developing six new realistic moral dilemmas, which we also use to validate our new self-report measures of the process of moral decision-making.

In the remainder of this article, we describe how we developed a new scale for measuring two important aspects of how a person made a specific moral decision: (1) reliance on moral rules versus CBR, and (2) reliance on deliberation versus intuition. We also introduce the six new moral dilemmas we used to validate these subscales.

## Development and evaluation of measures of reliance on different decision mechanisms

We developed and validated self-report subscales for measuring key aspects of how a person made a specific moral decision. The Reliance on Intuition versus Deliberation subscales measure how much the decision was driven by intuitive versus deliberative processing. The Reliance on Rules versus Consequences subscales measure how much the person relied on CBR versus moral rules (see Table 1).

We developed and psychometrically evaluated these measures in parallel over four studies. In Study 1, we collected data on people’s responses to initial items about their reliance on intuition versus deliberation. Each participant first made a decision in a moral dilemma and then rated their decision process according to the initial items. We then conducted an *exploratory factor analysis* (EFA) of these items and revised the subscales accordingly. In Study 2, we conducted a *confirmatory factor analysis* (CFA) of the finalized scale items on the Reliance on Intuition versus Deliberation subscales using six novel moral dilemma vignettes (more information about these vignettes in Section “Development of six new moral dilemmas’’). We also added a new initial item set of how much they relied on rules versus consequences, and conducted an EFA of these items. In Study 3, we conducted a CFA of the finalized scale items for the Reliance on Rules versus Consequences subscales using our novel vignettes. In Study 4, we tested the construct and predictive validity of our new subscales by investigating the relationship with existing scales and testing whether they predict choices in moral dilemmas above and beyond these existing scales. Finally, in Study 5, we confirmed the construct validity of the Reliance on Intuition and Deliberation Subscale by correlating it with an existing decision-making preference scale.

### Study 1: Reliance on intuition versus deliberation in moral decision-making

Study 1 tested subscales for measuring reliance on intuition versus deliberation. Examining the factor structure of the subscale for measuring reliance on intuition versus deliberation allowed us to determine whether relying more on deliberation necessarily means relying less on intuition or whether people’s reliance on each mechanism varies independently of their reliance on the other.

#### Method

##### Development of the initial item pool: intuition versus deliberation

In this section, we outline the process for deriving the items for reliance on intuition versus deliberation. A full list of the initial items can be found in Appendix A1.1. When developing the initial item set, we first considered other studies that have previously asked people about their decisions regarding specific moral dilemmas using ad hoc measures. We based some of our items that measure intuition and deliberation (items ID1 – ID4) on Oktar et al. (2023). In this study, participants saw some moral dilemmas and completed some “metacognitive scale items’’: *“Is your judgment based on deliberation/analysis?’’* and *“Is your judgment based on intuition/emotion?’’* As deliberation/analysis and intuition/emotion might be perceived as different concepts, we decided to keep these items separate into four separate questions. We also renamed “analysis’’ to “cost–benefit reasoning’’ as this was a better fit for the type of analysis that participants would do when considering moral dilemmas.

Further, we chose specific phrases from the domain-specific version of The Unified Scale to Assess Individual Differences in Intuition and Deliberation (items ID5 – ID7, USID, Pachur and Spaar, 2015), such as “gut feeling’’ and “use my heart as a guide’’. This domain-specific extension of the USID was developed to measure people’s domain-specific decision-making tendencies for six separate domains (mate choice, clothing, restaurants, medical, electronics, and vacation). However, this scale still asks people to report on their general tendencies across all of their decisions in a given domain. By contrast, our goal is to measure how the person made a specific individual decision. Therefore, we only selected specific phrases and heavily adapted them to fit the purposes of our measurement.

To measure deliberation (items ID8 – ID13), we did not think that the domain-specific USID items on deliberation were suitable for measuring deliberation in moral decision-making as they focused on planning and preference for complexity. Therefore, we developed new scale items to measure reliance on deliberation in moral decision-making through discussions with each other and by consulting the extant literature on moral decision-making.

##### Participants

This experiment received ethical approval from the Independent Ethics Commission of the Medical Faculty of the University of Tübingen under protocol #429/2024BO2.

We recruited 300 participants between the 23rd and 24th of June 2023. Each participant was paid £2.48 for their time. We excluded three participants who did not pass one of our two attention checks, which instructed them to put the slider of certain questions to 0 or 100. Therefore, our final sample contained 297 participants. The mean age was 43.2 years ($SD = 14.0$); 149 participants were female, and 148 were male.

##### Materials and procedure

We showed participants one of 13 randomly selected moral dilemmas from Maier et al. (2025), which were adapted from vignettes used in Körner and Deutsch (2023). After reading the moral dilemma, participants saw the items about reliance on intuition and deliberation.^1^ The statements were presented in randomized order. Participants were asked to what extent they agreed with each statement and gave their responses on a scale from 0 (“Not at all’’) to 100 (“Entirely’’). Each participant rated all items.

#### Results

##### Unique variable analysis: reliance on intuition versus deliberation

We first conducted a unique variable analysis (UVA, Christensen et al., 2023) to remove any redundant items. This is a crucial step in scale development, as selection solely based on reliability can otherwise lead to a scale in which the different items are very similar duplicates of each other. Consequently, the scale would only ask about a small subset of the construct in question. UVA has been shown to outperform similar methods for local independence detection in a large simulation study (Christensen et al., 2023). Based on the UVA, we removed the items ID7 (which was redundant with ID2) and ID13 (which was redundant with ID1 and ID11). When applying UVA to remove redundant items, we always selected the item that best covered the constructs of interest based on our theoretical judgment (i.e., the method identifies sets of items that are redundant with each other, and the researchers then decide which of the redundant items to retain).

##### Exploratory factor analysis: Reliance on intuition versus deliberation

Second, we ran an exploratory factor analysis (EFA) using the fa function in the psych package (Revelle, 2025) in R. We first tried to extract a one-factor solution, viewing the items about intuition as reverse-coded items. The single-factor solution showed a very poor fit (RMSEA $$= 0.172$$, TLI$$ = 0.431$$). This very poor fit was also retained when running a confirmatory factor analysis (CFA), which only selected the items that loaded more than .4 on the factor (RMSEA$$ = 0.193$$, TLI$$ = 0.656$$). Therefore, we examined a two-factor solution with intuition and deliberation as two orthogonal factors using varimax rotation, based on previous work considering intuition and deliberation as orthogonal mechanisms that may operate at the same time (Pachur & Spaar, 2015; Evans, 2008). The two-factor solution showed a better, though still poor, model fit (RMSEA$$ = 0.087$$, TLI $$= 0.854$$).

Thus, we proceeded to examine a solution where we only kept the three best items (in terms of the difference between loadings on the primary and secondary factors). All the items retained in this way had loadings larger than 0.5 on the primary factor and smaller than 0.2 on the other factor. This solution had a very good fit (RMSEA$$ = 0.041$$, TLI$$ = 0.980$$). Finally, we also compared this result to the result obtained using a non-orthogonal rotation (oblimin). This led to similar results, except for ID12 being included instead of ID1 for the intuition factor; however, we decided against this solution as item ID12 is inherently a trade-off item rather than an item measuring intuition or deliberation in isolation (“To what extent did you prioritize your intuition over rational thinking when making your decision?’’). As shown in Table 1, we retained six items in our final item set based on the orthogonal rotation.

### Study 2

In Study 2, we conducted a confirmatory factor analysis (CFA) of the finalized scale items developed in Study 1 using six new moral dilemma vignettes (see Section “Development of six new moral dilemmas’’) to ensure their generalizability. We also added a new initial item set of how much participants relied on rules versus consequences and conducted an EFA for these items.

#### Method

##### Participants

This experiment received ethical approval from the UCL Psychology Ethics Committee under code EP/2018/005. We recruited 300 participants on August 14, 2023. One participant did not pass one of the two attention checks. Therefore, our final sample contained 299 participants (149 female and 150 male), with a mean age of 41.6 ($SD = 13.9$). Each participant received a reimbursement of £2.50.

##### Materials and procedure

In Study 2, we showed participants a different set of six moral dilemma vignettes. Again, to dissociate the decision process (i.e., rules versus CBR) from the distinction between action versus omission, we had three dilemmas where the rule option was the choice action (“Rule Action’’) and three dilemmas where the CBR option was the choice action (“CBR Action’’). The full vignettes are included in Appendix A1.4. Participants were randomly assigned to read two moral dilemmas: one CBR Action and one Rule Action. They gave ratings on all the items after reading each dilemma.

##### Addition of new scale items: Reliance on rules versus consequences

To address a different component of moral decision-making, in Study 2 we developed new items to measure people’s reliance on following rules versus directly considering the consequences. We developed an initial item pool through discussions with each other and by consulting extant literature (e.g., Bennis et al., 2010; Gigerenzer and Brighton, 2009; Greene, 2013). A full list of the initial items for the measure of reliance on rules versus consequences can be found in Appendix A1.1.

#### Results

##### Confirmatory factor analysis: Reliance on intuition versus deliberation

We conducted a CFA of finalized items on intuition versus deliberation from Study 1 to test those items on a new sample. A CFA on the data from the CBR Action dilemmas indicated a good model fit (CFI$$ = 0.98$$, SRMR$$ =.058$$, RMSEA$$ =.066$$). For Rule Action dilemmas, the model fit was good according to the CFI and SRMR fit indices and ‘marginal’ according to the RMSEA (CFI$$ = 0.97$$, SRMR$$ =.043$$, RMSEA$$ =.096$$). While the RMSEA of 0.096 exceeds the conventional threshold of 0.08 for acceptable fit, this value may overestimate misfit in models with low degrees of freedom (e.g., Kenny et al., 2015). Considering that our model has $$df = 8$$, and that the other fit indices suggested acceptable model fit (especially SRMR, which is often preferable over RMSEA; Maydeu-Olivares et al., 2018), we retained the original model structure.

##### Unique variable analysis and exploratory factor analysis: reliance on rules versus consequences

We first conducted a UVA, removing items that were redundant for both CBR Action and Rule Action dilemmas. Based on this, we excluded items RC1, RC2, and RC8. We then conducted an EFA. We first tried a single-factor solution, but this solution only extracted one factor about the importance of following rules. We, therefore, next tried a two-factor solution. Consistent with the CNI model (Gawronski et al., 2017a), this approach extracted one factor about the importance of following rules and one factor about the importance of choosing actions that lead to the best consequences. Using the same approach as for the intuition versus deliberation items, we then retained the three highest loading items for each factor, resulting in a reliable scale to measure reliance on rules versus cost–benefit reasoning for both the CBR Action (CFI$$ = 1.00$$, RMSEA$$ =.000$$) and the Rule Action dilemmas (CFI$$ = 1.00$$, RMSEA$$ =.000$$). Both varimax and oblimin rotation would lead to the same choice for the items to be extracted. Thus, we retained six items for measuring reliance on rules versus consequences (see Table 1).

### Study 3

In Study 3, we conducted a CFA of the finalized scale items for rules versus consequences using the same set of novel vignettes used in Study 2.

#### Method

##### Participants

This experiment received ethical approval from the UCL Psychology Ethics Committee under code EP/2018/005. We recruited 300 participants on September 4, 2023. All participants passed the attention check. A total of 150 participants were female, and 150 were male. The mean age was 42.6 ($SD = 12.5$). Participants received £0.65 to participate in our 4-min study.

##### Materials and procedure

We used the same moral dilemma vignettes and procedure as in Study 2 (full materials in Appendix A1.4) and showed participants the scale items on reliance on rules versus consequences. Each participant saw one randomly selected dilemma and gave responses on the subscales.

#### Results

##### Confirmatory factor analysis: Reliance on rules versus consequences

We tested the reliability of the derived scales again in a new sample. This indicated a good model fit for the CBR Action dilemmas (CFI$$ = 1.00$$, SRMR$$ =.020$$, RMSEA$$ =.000$$) as well as for the Rule Action dilemmas (CFI$$ = 0.98$$, SRMR$$ =.036$$, RMSEA$$ =.081$$). Consistent with previous research on the processes underlying moral decision-making (e.g., Conway and Gawronski, 2013; Gawronski et al., 2017a), these findings suggest that reliance on rules and reliance on consequences are two separate dimensions of the process of moral decision-making.

### Study 4: Construct validity and predictive validity

Having established the reliability of our subscales, we now turn to their construct and predictive validity. To achieve this, Study 4 obtained three types of evidence for the construct validity of our subscales. First, we wanted to test discriminant construct validity by showing that the first pair of subscales (reliance on rules versus CBR) measures different constructs than the second pair of subscales (reliance on intuition versus deliberation).^2^ Second, to establish convergent construct validity, we tested if our subscales relate to the Deontology Subscale of the Deontological-Consequentialist Scale (DCS, Mata et al., 2022) and the Sacrificial Harm Subscale of the Oxford Utilitarianism Scale (OUS, Kahane et al., 2018). Third, to assess predictive validity, we tested whether our subscales could predict participants’ decisions in moral dilemmas beyond those predicted by existing scales.

To establish the convergent construct validity of our subscales for measuring people’s reliance on CBR versus rules, we measured their associations with the degrees to which people endorse the two conceptually most closely related moral theories, namely utilitarianism and deontology. To measure utilitarianism, we selected the OUS Sacrificial Harm Subscale, which is a well-known and commonly used measure of utilitarian beliefs. It specifically measures people’s willingness to inflict sacrificial harm when it is outweighed by other benefits, which is the decision that people are faced with in our dilemmas and a signature of CBR. To measure deontology, we selected the DCS Deontology Subscale because it is the logical counterpart to the OUS and maps onto the rule choice in moral dilemmas.

Based on the philosophical definitions of utilitarianism and deontology as discussed in the introduction of this article, one would expect reliance on CBR to be positively correlated with the OUS Sacrificial Harm Subscale and negatively correlated with the DCS Deontology Subscale. On the other hand, reliance on rules would likely be positively correlated with the DCS Deontology Subscale and negatively correlated with the OUS Sacrificial Harm Subscale. Assuming that reliance on CBR, reliance on rules, reliance on intuition, and reliance on deliberation are four separate constructs, one would not expect that reliance on deliberation versus intuition would be related to utilitarianism versus deontology in the same way.

#### Methods

Study 4 was a re-analysis of data originally collected for a different purpose. We re-analyzed data from Experiment 2 of Maier et al. (2025) to answer questions that are orthogonal to the hypotheses about moral learning tested in that article.

##### Participants

The sample size was $N=380$ ($\text {M}_\text {age} = 42.9$, $\text {SD}_\text {age} = 13.2$; 192 female and 188 male).

##### Materials and procedure

In Experiment 2 of Maier et al. (2025), participants saw a series of moral dilemmas where they had to choose between two conflicting options: one that was endorsed by CBR, and one that was endorsed by a moral rule. They did not rate any scale items in this process, and only made judgments about what they would do in each dilemma. Subsequently, participants read a Rule Action and a CBR Action dilemma that we developed and used in Studies 2 and 3 of this paper (see Section “Development of six new moral dilemmas’’). After reading each dilemma, participants completed our self-report measures of reliance on rules, CBR, intuition, deliberation in a new specific moral decision.^3^ They also gave responses to the DCS Deontology Subscale and the OUS Sacrificial Harm Subscale. More details about the procedure of this experiment are available in Maier et al. (2025). We include the responses to our scale items and the two abovementioned subscale items from that experiment in Study 4 of this paper.

#### Results

##### CBR versus deliberation and rules versus intuition are separate constructs

Studies 1–3 showed that reliance on rules and reliance on CBR are two separate factors, and that reliance on intuition and deliberation are two separate factors. However, because of how the subscales were distributed across different studies, we were unable to assess whether reliance on rules is conceptually distinct from reliance on intuition and whether reliance on CBR is conceptually distinct from reliance on deliberation. To answer each of these questions, we first conducted CFAs with a two-factor model and a one-factor model, and then compared the fit of both models. In general, in Study 4, we averaged across Rule Action and CBR Action dilemmas for analyses focusing on factor structure and reliability, but treated them separately when predicting participants’ choices.

The degrees to which participants reported relying on rules versus intuition were extremely well captured by a measurement model with two independent factors (CFI $= 1.00$, SRMR $= 0.019$, RMSEA $= 0.046$) and very badly captured by a measurement model with only a single factor (CFI $= 0.681$, SRMR $= 0.212$, RMSEA $= 0.386$). Consequently, a model comparison provided substantial evidence for the two-factor model explaining the data better than the one-factor model ($\text {BIC}_{two} = 22740$, $\text {BIC}_{one} = 23238$, $\text {AIC}_{two} = 22688$, $\text {AIC}_{one} = 23191$).

For reliance on CBR versus deliberation, we found an acceptable fit of the two-factor model (CFI$$ = 0.95$$, SRMR$$ = 0.053$$, RMSEA$$ = 0.122$$, for a more detailed discussion of results with high RMSEA but good CFI and SRMR see CFA in Study 2.) and a poor fit of the one-factor model (CFI$$ = 0.82$$, SRMR$$ = 0.088$$, RMSEA$$ = 0.209$$). Again, a model comparison provided substantial evidence for the two-factor model explaining the data better than the one-factor model ($$\text {BIC}_{two} = 22191$$, $$\text {BIC}_{one} = 22290$$, $$\text {AIC}_{two} = 22140$$, $$\text {AIC}_{one} = 22243$$).

The results of the model comparisons between two-factor and one-factor models suggested that reliance on CBR is distinct from deliberation, and that reliance on rules is distinct from reliance on intuition. The finding that they are distinct psychological constructs is positive evidence for the discriminant construct validity of our measures.

##### Reliance on CBR, rules, intuition, and deliberation are four separate constructs

Taken together, the CFA reported above suggests that the four subscales we developed measure four separate aspects of the (moral) decision-making process. To further strengthen the evidence for this four-factor model, we conducted a *parallel analysis* (Horn, 1965) followed by an EFA. We used the default settings for the fa.parallel method of the psych package (i.e., minimum residual solution and 20 iterations). The scree plot suggested four factors or three factors (see Fig. 2). Therefore, we conducted EFA for both a three-factor model and a four-factor model using oblimin rotation.

The four-factor model recovered the factor structure with items measuring reliance on rules, intuition, deliberation, and CBR, each loading on separate factors (see Table 7). This four-factor model fitted the data substantially better than the three-factor model, which had to assume that items measuring reliance on CBR and items measuring reliance on deliberation load on the same factor (four-factor: RMSEA $= 0.069$, BIC $= -75.02$; three-factor: RMSEA $= 0.098$, BIC $= -41.46$; see Table 8).^4^ These findings provide strong evidence for the structural validity of our four new subscales. The relationships between the items from all four subscales strongly support the four-factor structure we assumed by grouping them into four separate subscales measuring reliance on CBR, reliance on rules, reliance on intuition, and reliance on deliberation.

##### Relationships with existing scales provide evidence of convergent validity

To test the relationship with existing scales, we fitted a single six-factor model with one factor for each of our four newly developed subscales, one factor for the OUS Sacrificial Harm Subscale, and one factor for the DCS Deontology Subscale. We then inspected the correlations between the latent variables in this model. The six-factor model achieved a good fit to the data from the six corresponding questionnaires (CFI $$= 0.94$$, SRMR$$ = 0.061$$, RMSEA$$ = 0.059$$). Table 2 displays the correlations between the different subscales. We can see that reliance on CBR relates most strongly to the OUS Sacrificial Harm Subscale, whereas reliance on rules correlates most strongly with the DCS Deontology Subscale. Further, we found a strong negative correlation between reliance on CBR and reliance on rules, which is unsurprising given that these are usually considered two competing processes in moral judgment (typically studied as utilitarianism versus deontology and used as two conflicting choices in moral dilemmas in psychology experiments, e.g., Awad et al., 2018; Körner and Deutsch, 2023).

Consistent with the dual-process theory of morality (Greene, 2013), we found a strong positive correlation between reliance on CBR and deliberation. In addition, we observed a moderate negative correlation between reliance on rules and reliance on intuition. This is in conflict with certain interpretations of an early version of the dual process theory of morality, according to which rule decision-making is commonly based on intuition (Greene et al., 2008). However, proponents of the theory have acknowledged that this is not always the case, noting that rule morality sometimes relies on reasoning, a phenomenon called “rationalist deontological philosophy’’, which is “best explained as a rationalization of evolved emotional intuition’’ (Greene, 2007, p. 72). In other words, rule-based moral *reasoning* is a form of reasoning, regardless of where the rules come from. Consistent with our findings, this interpretation of Greene’s dual-process theory predicts that rule-based moral *reasoning* should be *negatively* correlated with intuition. Moreover, our finding is consistent with the idea that “going with the strongest intuition’’ is a competing alternative to “the conscious application of a moral rule or principle’’ proposed in Haidt’s social intuitionist theory (Haidt, 2001). Overall, these associations between our scale measures and self-report measures of people’s moral theories support their convergent construct validity.

**Table 2:** Correlations between scales

	CBR	Deliberation	Rules	Intuition	OUS-SHS	DCS-DS
CBR	1.00					
Deliberation	0.68***	1.00				
Rules	-0.50***	-0.03	1.00			
Intuition	0.37***	0.11	-0.31***	1.00		
OUS-SHS	0.35***	0.11	-0.42***	0.16	1.00	
DCS-DS	-0.17**	-0.04	0.47***	-0.09	-0.55***	1.00

*Note.* OUS-SHS refers to the Oxford Utilitarianism Scale Sacrificial Harm Subscale and DCS-DS refers to the Deontology-Consequentialist Scale Deontology Subscale. ** denotes $p < .01$ and *** denotes $p < .001$

**Table 3:** Beta coefficients and 95% CI when predicting the probability of choosing the CBR option from all six subscales in a *single* regression model, depending on whether action under consideration was the CBR option (“CBR Action’’) or the rule option (“Rule Action’’)

Scale	$\beta$ [95% CI]	
	CBR Action	Rule Action
CBR	1.45 [0.96, 1.93]***	1.58 [0.98, 2.18]***
Deliberation	$-0.04$ [$-0.41$, 0.34]	$-0.14$ [$-0.58$, 0.30]
Rules	$-2.03$ [$-2.51$, $-1.56$]***	$-2.35$ [$-2.90$, $-1.79$]***
Intuition	$-0.32$ [$-0.67$, 0.02]	$-0.11$ [$-0.54$, 0.32]
OUS-SHS	0.33 [$-0.02$, 0.67]	0.15 [$-0.23$, 0.52]
DCS-DS	0.16 [$-0.21$, 0.52]	0.36 [$-0.05$, 0.78]

*Note.* OUS-SHS refers to the Oxford Utilitarianism Scale Sacrificial Harm Subscale and DCS-DS refers to the Deontology-Consequentialist Scale Deontology Subscale. ** denotes $p < .01$ and *** denotes $p < .001$. The dependent variable was coded so that higher values on the predictor indicate a higher probability of choosing the CBR option. The results are independent of the order in which the predictors are added to the model

##### Predictive validity for moral decisions

Finally, we tested how well our new subscales explain decision-making in moral dilemmas. To do so, we used a logistic regression predicting participants’ choices in moral dilemmas (both CBR Action and Rule Action dilemmas, see Section “Development of six new moral dilemmas’’) from the mean scores of our four subscales as well as the OUS Sacrificial Harm Subscale and the DCS Deontology Subscale.^5^ Table 3 shows the results of this analysis. We found that when testing all the scales in the same model, participants’ self-reported reliance on CBR correctly predicted that they chose the CBR option over the rule option. Moreover, participants’ self-reported reliance on rules strongly predicted that they chose the rule option over the CBR option. When we controlled for these two key predictors, none of the other self-report measures had a significant effect on participants’ choices. Notably, the lower bound of the confidence intervals on rules and CBR is higher than the upper bound on any of the other four predictors (Table 3), suggesting that these coefficients predict better than any of the other coefficients.

We found that only reliance on rules and reliance on CBR were predictive of choices in a model including all six subscales. This raises the question of whether the other scales simply do not predict behaviour well, or whether they merely do not have any incremental validity once reliance on rules and CBR is already taken into account. To answer this question, we also fitted six regression models that predicted choices from each of the six subscales *separately*. As shown in Table 4, all of our new subscales can be used to predict people’s decisions in moral dilemmas (except that reliance on intuition did not significantly predict people’s behavior in CBR Action dilemmas, $z = 1.24, p =.214$). The OUS Sacrificial Harm Subscale and the DCS Deontology Subscale also explained people’s choices.

We also tested whether reliance on intuition versus deliberation predicted participants’ response times. This did not show a significant relationship (for more details see Appendix A1.3). This is unsurprising given that response time analyses are more suited for experiments with shorter trials, whereas in our task, participants read a substantial amount of text in each trial, which likely makes reading and understanding speed a bigger influence on response times than the decision process (Van Zandt & Townsend, 2013; Donkin & Brown, 2018; Baron & Gürçay, 2017).

**Table 4:** Beta coefficients and 95% CI for predicting the probability of choosing the CBR option from each of the six subscales individually in *separate* regression models

Model with each subscale	$\beta$ [95% CI]	
	CBR Action	Rule Action
Model with only CBR	1.52 [1.18, 1.85]***	1.54 [1.21, 1.87]***
Model with only Deliberation	0.34 [0.13, 0.56]**	0.39 [0.17, 0.60]***
Model with only Rules	$-1.96$ [$-2.34, -1.58$]***	$-2.24$ [$-2.66, -1.81$]***
Model with only Intuition	0.13 [$-0.07, 0.34$]	0.69 [0.45, 0.93]***
Model with only OUS-SHS	0.60 [0.37, 0.82]***	0.52 [0.30, 0.74]***
Model with only DCS-DS	$-0.55$ [$-0.78, -0.33$]***	$-0.42$ [$-0.64, -0.22$]***

*Note.* OUS-SHS refers to the Oxford Utilitarianism Scale Sacrificial Harm Subscale and DCS-DS refers to the Deontology-Consequentialist Scale Deontology Subscale. *** denotes $p < .001$. The dependent variable was coded so that higher values on the predictor indicate a higher probability of choosing the CBR choice

Overall, the results presented in this section showed that (1) all subscales had high predictive validity for people’s moral decisions, (2) the subscales measuring reliance on CBR and rules predicted choices the best, and (3) once these subscales were accounted for, there was no evidence that the other validated scales provided additional information about people’s decisions in sacrificial moral dilemmas.

### Study 5: Construct validity of the reliance on intuition versus deliberation subscales

We conducted an additional study to test the construct validity of the Reliance on Intuition versus Deliberation subscales by correlating responses with the Preference for Intuition and Deliberation (PID) Scale (Betsch, 2004), a validated scale on people’s general preferences for intuitive versus deliberative decision-making. The PID was designed to measure decision-making preferences that are more stable over time (i.e., as an individual difference measure), whereas our subscales were designed to measure decision processes in specific choices. Nevertheless, we would expect some correlation between a general preference for deliberation (intuition) and participants’ inclination to rely on deliberation (intuition) in a specific dilemma.

We preregistered the following predictions: our Reliance on Intuition subscale will correlate positively with Preference for Intuition as measured by the PID (H1), and our Reliance on Deliberation subscale will correlate positively with Preference for Deliberation as measured by the PID (H2). The experiment was preregistered at https://osf.io/7s3hv.

#### Method

##### Participants

We recruited $N = 400$ UK participants from Prolific on August 5, 2025. One participant failed the attention check, leaving a final sample size of $N=399$ 200 participants were female and 199 were male. The mean age was 41.2 ($SD =$ 13.3).

Materials. We showed participants one randomly selected moral dilemma (out of the six moral dilemmas we developed and used in previous studies). After reading the dilemma and making a decision, they completed the Reliance on Intuition versus Deliberation Subscales. Participants either completed the PID Scale after this or at the very beginning of the experiment (prior to reading the moral dilemma). All scale items were presented in randomized order. As an attention check, we asked participants to move the slider to 100 on a specified item.

##### Data analysis

We used the same approach as for the CFA in the previous study, fitting a structural equation model with the four subscales, and inspected the correlations between the latent variables. We further preregistered to check the robustness of our results using correlations of mean scores of model fit, which was poor (RMSEA $>.10$ or CFI $<.95$).

#### Results

As predicted, we found a positive correlation between our Reliance on Intuition Subscale and the PID-Intuition Subscale, $r = 0.63, p <.001$, and a positive correlation between our Reliance on Deliberation Subscale and the PID-Deliberation Subscale, $r = 0.45, p <.001$.

The four-factor model achieved a poor fit to the data, RMSEA$$= 0.063$$, SRMR $$=.067$$, CFI$$ = 0.846$$.^6^ As preregistered, since the model fit was poor (CFI$$ <.95$$), we also analyzed the correlations using mean scores. We found similar results in the analysis using mean scores (see Table 5). Importantly, the poor model fit was due to the inclusion of the PID scale; when fitting a model using only our two subscales, the model fit was excellent (RMSEA$$= 0.019$$, SRMR $$=.029$$, CFI$$ = 0.996$$).

**Table 5:** Correlation matrices between Reliance on Intuition and Deliberation Subscales and the PID scale, based on **A** latent variables and **B** mean scores (95% CI)

(A) Correlations based on latent variables				
	Intuition	Deliberation	PID-Intuition	PID-Deliberation
Intuition	1.00			
Deliberation	0.02 [$-0.10$, 0.15]	1.00		
PID-Intuition	0.63$$^{***}$$ [0.47, 0.79]	0.09 [$$-0.03$$, 0.22]	1.00	
PID-Deliberation	$$-0.10$$ [$$-0.22$$, 0.02]	0.45$$^{***}$$ [0.30, 0.61]	0.06 [$$-0.06$$, 0.18]	1.00
(B) Correlations based on mean scores				
	Intuition	Deliberation	PID-Intuition	PID-Deliberation
Intuition	1.00			
Deliberation	$-0.01$ [$-0.11$, 0.09]	1.00		
PID-Intuition	0.49$$^{***}$$ [0.41, 0.56]	0.06 [$$-0.04$$, 0.15]	1.00	
PID-Deliberation	$$-0.04$$ [$$-0.14$$, 0.05]	0.34$$^{***}$$ [0.25, 0.43]	0.11$$^{*}$$ [0.01, 0.20]	1.00

*Note.* ‘Intuition’ and ‘Deliberation’ refer to the Reliance on Intuition and Deliberation subscales we developed. ‘PID-Intuition’ and ‘PID-Deliberation’ refer to the subscales of the Preference for Intuition and Deliberation (PID) scale. * $p < .05$, ** $p < .01$, *** $p < .001$. *Lower triangle shown for clarity*

Overall, the results of Study 5 provide evidence for the construct validity of the Reliance on Intuition and Reliance on Deliberation subscales. They correlate with measures that we expected them to correlate with (general preference for intuition and deliberation), whereas we found no evidence for a correlation with other scales.

**Fig. 1:**
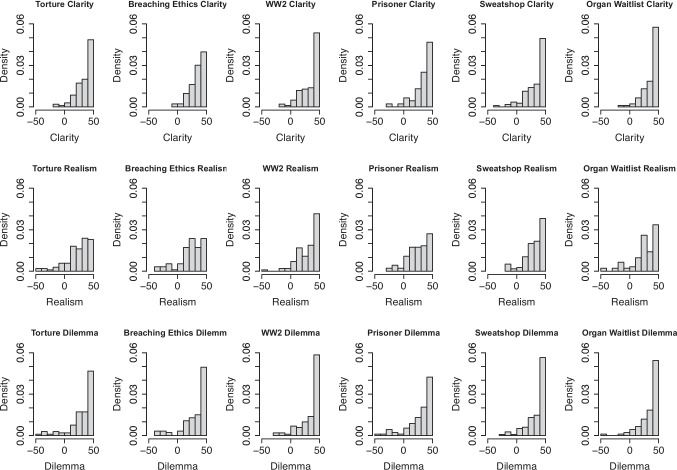
Participants rated all six moral dilemma vignettes as being very clear, realistic, and constituted a moral dilemma

## Development of six new moral dilemmas

We extended upon the work of Körner and Deutsch (2023) and Maier et al. (2025) by developing six new realistic trolley-type dilemma vignettes. In half of these dilemmas, the action under consideration is the CBR option (“WW2’’, “Torture’’, and “Breaching Ethics’’), and in the other half it is the rule option (“Organ Waitlist’’, “Sweatshop’’, and “Prisoner’’). As an example of a CBR Action dilemma, in “WW2’’, the reader is asked to imagine themselves as a soldier fighting the Nazis in World War II. They need to decide whether to proceed with an attack on an enemy outpost. If they decide to proceed with the attack, they would probably harm some innocent civilians in the vicinity, but the attack would weaken the enemy’s capabilities and potentially end the war sooner, saving many more innocent people. Thus, proceeding with the attack is the CBR option, and not doing so is the rule option. As an example of a Rule Action dilemma, in “Organ Waitlist’’, the reader is asked to imagine themselves as a surgeon who needs to decide whether to follow the organ donation waiting list and give the only available donor heart to an older patient who is next on the list, or not follow the waiting list and give it to a younger patient with a higher chance of survival. Here, following the waiting list is the rule option, and not doing so is the CBR option, as it breaks the moral rule of fairness, but could potentially save many more years of a person’s life. Full materials for this and all other vignettes are available in Appendix A1.4.

We used these dilemmas to test our scale in Studies 2–5. Our dilemmas covered a variety of realistic scenarios that involved different rule violations (i.e., harming civilians, torture, disregarding established safety rules or fairness regulations, exploiting others, and killing) and different contexts (i.e., war/military, medicine, industry, and justice). Having tested our scale on such a wide range of moral decisions makes our findings about its reliability and validity more generalizable. We invite future studies using our scale to take advantage of these and other diverse moral dilemmas to increase the generalizability of their findings.

In Study 2, we asked participants ($N = 299$) to evaluate the quality of the dilemmas by asking them how clear it was (on a scale of -50 = “Extremely unclear’’, 0 = “Neutral’’, to 50 = “Extremely clear’’), how realistic it was (on a scale of -50 = “Extremely unrealistic’’, 0 = “Neutral’’, to 50 = “Extremely realistic’’), and how much it constituted a moral dilemma (on a scale of -50 = “Completely not a dilemma’’, 0 = “Neutral’’, to 50 = “Completely a dilemma’’). As shown in Fig. 1, participants rated the dilemmas as very clear and realistic and agreed that each of the situations constituted a moral dilemma.

## Conclusion

We developed the Decision Process Scale (DPS) that can be used to measure the processes by which people make moral decisions. We present a reliable scale to measure their reliance on intuition, deliberation, rules, and cost–benefit reasoning. This reliable self-report measure can help researchers gain a more fine-grained understanding of the decision processes people employ in specific scenarios.

Across our studies, we found a good fit of all our measures. Study 1 suggested that intuition and deliberation were two independent dimensions of moral decision-making, and Study 2 confirmed this conclusion. Likewise, Study 2 suggested that reliance on rules and reliance on CBR were two independent dimensions of moral decision-making, and Study 3 confirmed this conclusion. Study 4 confirmed the construct and predictive validity of our subscales, and Study 5 also showed good construct validity of the Reliance on Intuition and Deliberation Subscale.

Consistent with previous research on peoples’ decision-making styles (e.g., Pachur and Spaar, 2015; Betsch, 2008), these findings suggest that relying on intuition and deliberation are not mutually exclusive. Instead, they should be considered two independent contributors to decision-making. This means that some decision processes may employ both more intuition and more deliberation than others. Intuition and deliberation are not necessarily two competing alternatives, as was sometimes (implicitly) assumed in previous research on moral judgment and moral decision-making.

Likewise, consistent with previous findings on the processes underlying moral decision-making (e.g., Gawronski et al., 2017a), our findings suggest that people’s reliance on rule-based decision-making and cost–benefit reasoning is not a zero-sum game, either. Instead, people might be able to increase their reliance on either without having to decrease their reliance on the other. This adds to the recent finding that people integrate the recommendations of rules and cost–benefit reasoning when making decisions in moral dilemmas (Guzmán et al. (2022); see also Greene (2023)). Future research could attempt to relate our scales directly to process dissociation results (Gawronski et al., 2017a) and tasks to measure cognitive processes, such as the two-step task (Daw et al., 2011; Lockwood et al., 2016). However, these paradigms are not designed as individual difference measures, and we therefore caution researchers to use a large number of trials when measuring individual differences and even then only expect small effect sizes (Pedroni et al., year; Rey-Mermet et al., 2025; Körner et al., 2020).

With only 12 items and three items per dimension, the DPS is relatively short. Usually, adding additional items to a scale increases its reliability, but at the cost of increasing the time it takes to answer all questions. We envision that the DPS might be used as part of (moral) decision-making experiments involving multiple trials where different decisions need to be made. In this context, the brevity of the DPS is a strength, as it allows researchers to elicit these judgments after each dilemma without considerably increasing the overall length of the experiment. Future work could develop extended versions of the scale for other contexts, such as a task with only one dilemma or vignette.

While we tested the reliability of the DPS only for moral dilemmas, it may also be applicable in other contexts where rules and cost–benefit reasoning are in conflict. This includes situations where one decides whether to break the law, and situations where one chooses between relying on a heuristic decision process and an explicit cost–benefit analysis. Therefore, this work has the potential to improve the measurement of decision processes across a variety of areas in psychology, and not just moral psychology. While we cannot think of a good reason why the factor structure or the reliability of the scales should change in a related domain, researchers could easily re-establish the measure’s theoretic properties in a new domain by rerunning our confirmatory factor analysis on data from a different context.

In conclusion, the Decision Process Scale provides a robust tool for accurately measuring important facets of the decision processes individuals employ in moral dilemmas. We are hopeful that the application of these findings will facilitate scientific progress on these crucial topics.

## Figures and Tables

**Fig. 2:**
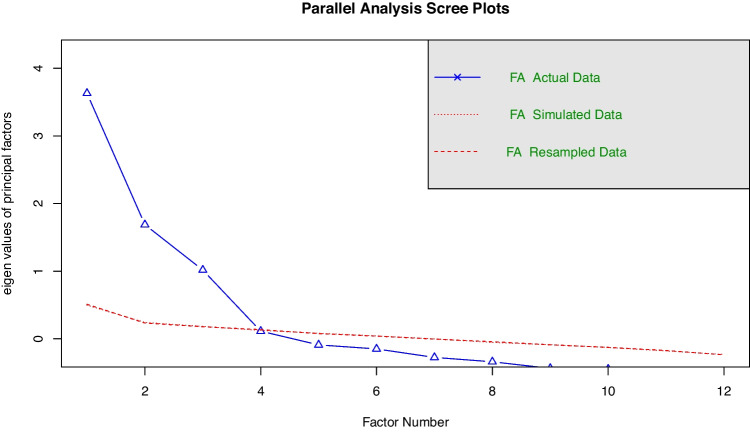
Scree plot for parallel analysis. *Note*. FA actual data $=$ eigenvalues of factor solutions based on the actual data. FA simulated data $=$ eigenvalues of factor solutions based on randomly simulated normally distributed data with the same sample size and number of items. FA resampled data $=$ eigenvalues of factor solutions of randomly resampled subsets of the actual data. The factor extraction decision is based on a comparison between the actual data and the random datasets, which here would indicate 3–4 factors

## Data Availability

Yes (in Appendix and https://osf.io/82cfz/)

## Appendix

### Initial item pool

#### Items on intuition versus deliberation

1. To what extent did you rely on your intuition when making your decision?
2. To what extent did you rely on your emotions when making your decision?
3. To what extent did you rely on deliberation when making your decision?
4. To what extent did you rely on cost–benefit reasoning when making your decision?
5. To what extent was it important that your decision felt right?
6. To what extent was it important that you had a rational reason for your decision?
7. To what extent did you rely on your heart as a guide for your actions when making your decision?
8. To what extent did you consider the anticipated consequences of your decision?
9. To what extent did you use logical reasoning when making your decision?
10. To what extent did you carefully evaluate the pros and cons of your decision compared to the alternative decision?
11. To what extent did you trust your gut feeling when making your decision?
12. To what extent did you prioritize your intuition over rational thinking when making your decision?
13. To what extent did you rely on your instincts when making your decision?

#### Items on rules versus consequences

1. To what extent was it important that the action you chose followed the rules?
2. To what extent was it important that the action you chose followed norms?
3. To what extent was it important that the action you chose did NOT violate any rules?
4. To what extent was it important that the action you chose did NOT violate any norms?
5. To what extent did you rely on rules when making your decision?
6. To what extent did you rely on norms when making your decision?
7. To what extent was it important that the action you chose would maximize overall happiness for everyone?
8. To what extent was it important that the action you chose would benefit the most people?
9. To what extent did you choose your action based on its anticipated consequences?
10. To what extent did you evaluate the anticipated consequences of the action that you did not choose?
11. To what extent was it important that the action you chose would have better consequences than the action you did not choose?

### Metacognitive level items on integration of moral decision-making processes

These items aimed to measure how much the person thought about arbitrating between or integrating the recommendations of using moral rules versus cost–benefit reasoning.

For instance, some people may just rely on a rule without explicitly thinking about whether to do so or not, whereas other people might explicitly think about how the relative reliability of moral rules and CBR depends on relevant features of the current situation.

#### Initial items

1. To what extent did you think about whether the anticipated benefits of breaking the rule are worth the moral cost?
2. To what extent did you think about how to combine the moral value of following the rule with the moral value of its consequences?
3. To what degree did you think about how to trade off the moral value of following the rule against the moral value of its consequences?
4. To what extent did you think about how to integrate the moral value of following versus breaking the rule with the moral value of its consequences?

#### Unique variable analysis and factor analysis

UVA indicated that I2 should be removed because it was redundant with I3 and I4. With only three items left, a CFA for a one-factor solution with items I1, I3, and I4 yielded a saturated model with zero degrees of freedom. Therefore, we did not go ahead with further inspection of fit indices. As shown in Table 6, we retained those three items.

**Table 6:** Items to measure reliance on integration

Integration
To what extent did you think about whether the anticipated benefits of breaking the rule are worth the moral cost?
To what degree did you think about how to trade off the moral value of following the rule against the moral value of its consequences?
To what extent did you think about how to integrate the moral value of following versus breaking the rule with the moral value of its consequences?

*Note.* We presented participants with the prompt “Please consider the following statements and think about to which extent you agree with each statement,’’ to which they indicated their agreement on a scale of 0 (“Not at all’’) to 100 (“Entirely’’)

### Study 4 supplementary results

#### Exploratory factor analysis

**Table 7:** Factor loadings for the four-factor model’s fit to the data from Study 4

Item	Rules	Intuition	CBR	Deliberation
Rules item 1	0.97	$-0.01$	0.03	0.01
Rules item 2	0.89	0.03	$-0.01$	0.02
Rules item 3	0.86	$-0.04$	$-0.06$	0.01
Intuition item 1	$-0.04$	0.91	$-0.04$	0.05
Intuition item 2	0.09	0.51	0.23	$-0.25$
Intuition item 3	0.01	0.91	0.00	0.00
Consequences item 1	0.02	0.03	0.72	$-0.12$
Consequences item 2	$-0.08$	0.02	0.58	0.17
Consequences item 3	$-0.22$	0.04	0.67	0.17
Deliberation item 1	0.03	0.01	$-0.09$	0.78
Deliberation item 2	0.07	$-0.01$	0.37	0.55
Deliberation item 3	0.04	0.06	0.27	0.55

*Note.* For full items, see Table 1

**Table 8:** Factor loadings for the three-factor model’s fit to the data from Study 4

Item	Rules	CBR/Deliberation	Intuition
Rules item 1	0.96	0.03	0.01
Rules item 2	0.90	0.01	0.04
Rules item 3	0.87	$-0.04$	$-0.03$
Consequences item 1	$-0.18$	0.42	0.16
Consequences item 2	$-0.18$	0.61	0.08
Consequences item 3	$-0.33$	0.67	0.11
Deliberation item 1	0.20	0.61	$-0.12$
Deliberation item 2	0.10	0.82	$-0.05$
Deliberation item 3	0.10	0.72	0.01
Intuition item 1	0.01	0.02	0.88
Intuition item 2	$-0.01$	$-0.05$	0.59
Intuition item 3	0.04	$-0.01$	0.92

*Note.* For full items, see Table 1.

#### Response times

To check whether reliance on intuition versus deliberation could predict response times in moral dilemmas, we fit an SEM model with a regression component predicting response times from those two subscales. The model showed acceptable model fit (RMSEA $$=.083$$, SRMR $$=.051$$, CFI = .96); however, we found no evidence that either reliance on intuition or reliance on deliberation predicted response times (Deliberation: $$b = -.006$$, z = 0.036, $$p = $$ .972, Intuition: $$b =.090$$, z = 1.124, $$p = $$ .261).

### Moral dilemma vignettes

#### “WW2’’ (CBR Action)

You are a military strategist fighting the Nazis during World War II. You lead a group of soldiers during a wartime operation. Your mission is to destroy a strategically important enemy outpost that poses a significant threat to your forces.

As you plan the attack, you discover that the outpost is located near a densely populated civilian area. Attacking the outpost would undoubtedly result in collateral damage, potentially causing harm to innocent civilians.

If you proceed with the attack, you would probably harm some innocent civilians in the vicinity, but the attack would weaken the enemy’s capabilities and potentially end the war sooner, saving many more innocent people. If you abort the mission, it would probably protect the nearby civilian lives, but allow the enemy outpost to remain intact, posing an ongoing threat to the people who are suffering under the Nazi regime.

Do you proceed with the attack? (Yes / No)

#### “Torture’’ (CBR Action)

You are a military commander stationed in Mali as part of a peacekeeping mission, and your unit has captured a member of a terrorist group who is believed to have crucial information about an impending attack on civilian targets within a densely populated city. Intelligence reports suggest that the enemy has planted explosives in several key locations, set to detonate in 24 h, with the potential to kill innocent civilians and cause extensive damage.

The captured terrorist is a high-ranking member of a notorious extremist group and has been identified as a mastermind behind several similar attacks in the past. Standard interrogation methods have failed to yield any information from him, and the clock is ticking. Members of your team, desperate to prevent the attack, have proposed resorting to torture as a last-ditch effort to extract the information needed to locate and defuse the bombs.

If you do not get the information and the attack goes ahead, innocent civilians will die. If you resort to torture, you are causing severe pain to a prisoner, violating their right to a fair trial, and committing a human rights violation.

Do you resort to torture? (Yes / No)

#### “Breaching ethics’’ (CBR Action)

You are a scientist working on a groundbreaking medical research project. Your team has developed a promising experimental treatment for a rare and currently incurable disease, which you want to test on a clinical sample of participants who have this disease.

However, the treatment approach violates certain ethical regulations that researchers are expected to comply with. In order to proceed with clinical trials, you need to bypass those regulations. If you proceed, the clinical trial might reveal the treatment to be safe and highly effective. If so, the treatment would likely be approved and become a standard procedure in hospitals around the world. However, without any further tests, it will continue to be prohibited.

If you choose to proceed with the experimental treatment and circumvent ethical guidelines, you may potentially provide a cure to thousands of patients and save countless lives if the treatment is effective; however, you risk your research participants’ lives and the scientific integrity of your research. If you choose to follow the established ethical regulations, you would be prioritizing safety, but you would also be delaying medical progress and denying many patients a chance for a treatment that might save their lives.

Do you breach the ethics regulations to proceed with the experimental treatment? (Yes / No)

#### “Organ waitlist’’ (Rule Action)

You are a surgeon specializing in organ transplantation. An elderly patient in critical condition urgently needs a heart transplant to survive. There is only one suitable donor heart available, and it matches another patient who is younger and has a higher chance of a successful outcome.

The younger patient is currently on life support and is expected to die soon without a transplant. However, the older patient is first on the waiting list for a new heart, and according to medical regulations, you ought to prioritize him.

If you give the heart to the younger patient, he has a higher chance of survival and a potentially longer life ahead, but this would disregard the waiting list, which was put in place to give everyone a fair chance at an organ transplant. If you instead honor the waiting list and give the heart to the older patient, the younger patient will lose many more years of life than the older patient, who also has many other serious health issues, could gain.

Do you follow the waitlist? (Yes / No)

#### “Sweatshop’’ (Rule Action)

You are the CEO of a large multinational corporation. One of your company’s factories, located in a developing country, has been found to be engaging in unethical labor practices, including underpaying workers and having unsafe working conditions.

In a report about the factory, you read that hundreds of workers are currently employed there and depend on their wages to support their families. However, you are also aware that by allowing the factory to continue operating, you are complicit in the ongoing exploitation and harm of vulnerable workers. Your employee asks you what you want to do in this situation, and whether you want to shut down the factory.

If you close the factory, you uphold ethical standards and protect workers’ rights. However, the livelihoods of your workers will be severely impacted, as they need the money and would either be unable to get another job or settle for one that is even worse. If you keep the factory open, you maintain the employment opportunities but perpetuate the exploitation of underprivileged workers.

Do you close the factory? (Yes / No)

#### “Prisoner’’ (Rule Action)

It is 1998 and you are a leader of a military troop in the Democratic Republic of Congo fighting against an enemy militant group. During a battle, you capture an important member of the militant group. This militant is responsible for ordering numerous attacks on civilian populations, resulting in the deaths of innocent people, including children. Since the intelligence service had already wiretapped this officer for a long time, he likely does not have any new information about the militant group that would be useful for you.

Soon after his capture, you receive news that the enemy is moving towards your camp and therefore you and your troops need to quickly retreat. You are only a small group of soldiers on enemy territory and therefore it is difficult to ensure the prisoner is guarded well - he is likely to escape during the retreat. Indeed, a prisoner had escaped in a similar situation before. Angered by the militant’s atrocious war crimes, your soldiers demand to execute the militant. They are on the way to the prisoners’ camp and will kill him if you do not intervene.

If you order your soldiers to stop, you can treat the militant as a prisoner of war and give him access to a fair trial later on. However, he may be able to escape, potentially leading to more future attacks and loss of innocent lives. If you allow your soldiers to execute him immediately, you ensure that he cannot harm anyone else, but you would be killing a defenseless prisoner without a trial and committing a human rights violation.

Do you order your soldiers to stop? (Yes / No)

## Footnotes

1 We also showed participants items about metacognitive integration between intuition and deliberation (see Appendix for more details).

2 The previous analyses only looked at the two-factor structure of reliance on CBR versus rules and the two-factor structure of reliance on intuition versus deliberation.

3 Participants also completed the scale for measuring the integration of these processes, but we did not include this scale in the re-analysis because it was not relevant to the aims of Study 4.

4 These results are based on the principal factor method, but are robust when using different factoring methods, such as minimum residual or weighted least squares. Further, we find the same results when using varimax instead of oblimin rotation.

5 We used logistic regression rather than SEM because the SEM did not converge, indicating that the covariance matrix of latent variables is not positive definite and that standard errors could not be computed. These convergence issues could not be addressed by switching to different estimators or a Bayesian SEM model using blavaan.

6 Because the items in the PID only range from 1 to 5, we also attempted modelling them as ordinal using polychoric correlations and estimator WLSMV (weighted least squares with mean variance adjusted test statistic). This still led to a poor fit, RMSEA$$= 0.068$$, SRMR $$= 0.076$$, CFI$$ = 0.870$$, and very similar results in terms of the latent correlations, with only the two predicted correlations being significant.

## Code availability

Yes (https://osf.io/82cfz/)

## Declarations

### Preregistration

Study 5 was preregistered (https://osf.io/7s3hv) prior to conducting the research, and the preregistration adheres to the disclosure requirements of the institutional registry.
